# Dosimetric Comparison of Dedicated Radiosurgery Platforms for the Treatment of Essential Tremor: Technical Report

**DOI:** 10.7759/cureus.57452

**Published:** 2024-04-02

**Authors:** Neelan J Marianayagam, Ian Paddick, Amit R Persad, Yusuke S Hori, Alexander Maslowski, Ishwarya Thirunarayanan, Arjun R Khanna, David J Park, Vivek Buch, Steven D. Chang, M. Bret Schneider, Hong Yu, Georg A Weidlich, John R. Adler

**Affiliations:** 1 Neurosurgery, Stanford University School of Medicine, Stanford, USA; 2 National Centre for Neurology and Neurosurgery, Queen Square Radiosurgery Centre, London, GBR; 3 Medical Physics, Zap Surgical Systems, Inc., San Carlos, USA; 4 Software Development, Zap Surgical Systems, Inc., San Carlos, USA; 5 Neurological Surgery, Stanford University Medical Center, Palo Alto, USA; 6 Stereotactic Radiosurgery, Zap Surgical Systems, Inc., San Carlos, USA; 7 Psychiatry, Stanford University School of Medicine, Stanford, USA; 8 Neurological Surgery, Vanderbilt University, Nashville, USA; 9 Radiation Oncology, Stanford University Medical Center, Palo Alto, USA

**Keywords:** neurosurgery, functional neurosurgery, stereotactic radiosurgery, essential tremor, movement disorders

## Abstract

Essential tremor (ET) is one of the most common adult movement disorders. As the worldwide population ages, the incidence and prevalence of ET is increasing. Although most cases can be managed conservatively, there is a subset of ET that is refractory to medical management. By virtue of being “reversible”, deep brain stimulation (DBS) of the ventral intermediate nucleus (VIM) of the thalamus is one commonly accepted intervention. As an alternative to invasive and expensive DBS, there has been a renaissance in treating ET with lesion-based approaches, spearheaded most recently by high-intensity focused ultrasound (HIFU), the hallmark of which is that it is non-invasive. Meanwhile, stereotactic radiosurgical (SRS) lesioning of VIM represents another time-honored lesion-based non-invasive treatment of ET, which is especially well suited for those patients that cannot tolerate open neurosurgery and is now also getting a “second look”. While multiple SRS platforms have been and continue to be used to treat ET, there is little in the way of dosimetric comparison between different technologies. In this brief technical report we compare the dosimetric profiles of three major radiosurgical platforms (Gamma Knife, CyberKnife Robotic Radiosurgery, and Zap-X Gyroscopic Radiosurgery (GRS)) for the treatment of ET. In general, the GRS and Gamma Knife were shown to have the best theoretical dosimetric profiles for VIM lesioning. Nevertheless the relevance of such superiority to clinical outcomes requires future patient studies.

## Introduction

Movement disorders refractory to medical management continue to incapacitate large numbers of patients worldwide. Furthermore, the relentless aging of the world population is significantly increasing the prevalence of these neurological diseases. Essential tremor (ET) can be among the most debilitating of all adult movement disorders. Treatment of ET begins with behavioral modification, physical therapy, and/or medical management [1-3]. However, a certain percentage of patients are refractory to such conservative management options, and more invasive interventions are deemed necessary. Unilateral or bilateral deep brain stimulation (DBS) of the ventral intermediate nucleus of the thalamus (VIM) can be a highly effective treatment [4,5], yet some patients are unable to tolerate or afford this invasive and costly neurosurgical procedure. In lieu of DBS, lesion-based approaches such as focused ultrasound to the VIM nucleus can also produce excellent clinical outcomes [6,7]. Of note, one noninvasive alternative, stereotactic radiosurgery (SRS) thalamotomy, has a track record of efficacy spanning several decades [8].

SRS thalamotomy has been proven to be a safe method for treating ET [8-10]. It has shown good results in a wide range of populations. Efficacy has been reported at between 69-75% of patients undergoing SRS thalamotomy for ET [8-10]. However, since the target for lesioning (VIM) is located in a sensitive area of the brain (thalamus), side effects such as contralateral motor symptoms have been experienced by 4-8% of patients undergoing SRS [8]. As such, more accurate targeting of this region with minimal dose spill-off is of utmost importance. In this paper we compare the dosimetric profiles for three major SRS platforms.

The three major modalities and treatment devices for SRS are cobalt-based units such as the Elekta Gamma Knife® (GK; Elekta Instruments, Atlanta, GA, USA), robotic radiosurgery (RRS) such as the Accuray CyberKnife® (Accuray, Inc., Sunnyvale, CA, USA), and gyroscopic radiosurgery (GRS) such as the ZAP Surgical ZAP-X® (ZAP Surgical Systems, Inc., San Carlos, CA, USA). Although there are differences in the technology between the three, generally speaking, all three have similar technical performance that has been compared systematically [11]. In this paper we describe and dosimetrically compare the technical aspects of these three modalities in the treatment of ET.

## Technical report

Target selection

Utilizing a single imaging data set from a patient whom had previously undergone DBS for ET, the VIM nucleus was delineated on a fast gray matter acquisition T1 inversion recovery (FGATIR) Magnetic Resonance Imaging (MRI) which had been algorithmically fused to a thin slice computerized tomography (CT) by means of the Accuray Precision® TPS treatment planning system (TPS). The FGATIR sequence MRI allows for anatomic localization of the VIM using the anterior commissure-posterior commissure (ac-pc) plane. This plane is delineated by a line joining the superior edge of the anterior commissure and the inferior edge of the posterior commissure [12]. In most subjects the center of VIM lies between 2-4 mm superior to the ac-pc plane. In the case of our sample patient and for purposes of this study 4 mm was chosen [12]. After the VIM of the thalamus was identified, the overall anatomic location was visually inspected to confirm that this position “made sense”. A gross target volume (GTV) was then drawn centered at the above coordinate, assuring that the internal capsule was outside the targeted volume. After performing the above target localization, the resulting DICOM RT structure set was shared among three different SRS platforms (GK, RRS, and GRS), each of which created treatment plans for a VIM lesion.

Robotic radiosurgery treatment planning

**Figure 1:**
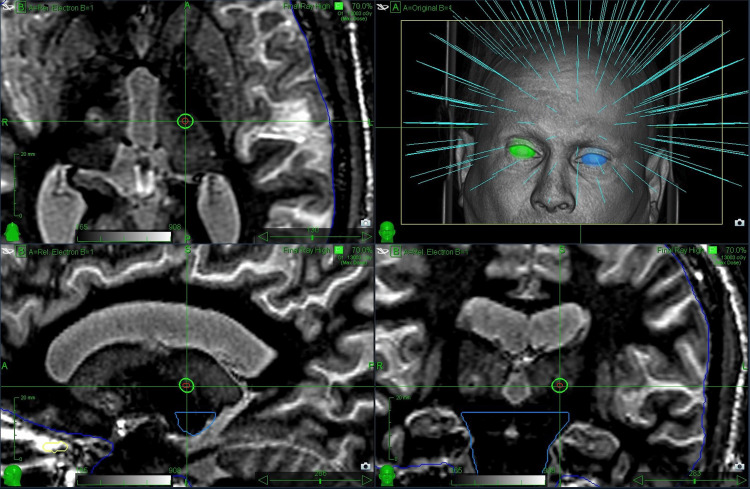
Robotic radiosurgery (RRS) treatment plan for essential tremor using a 5 mm collimator.

A Raytracing treatment plan with all beam trajectories was generated using a 5 mm diameter conical collimator and a prescription dose of 91 Gy as defined at the 70% isodose line. Of note, the nominal energy of the CyberKnife linear accelerator (LINAC) beam is 6 MeV with an source-to-axis distance (SAD)=80cm. The resulting treatment plan utilizing 153 intersecting beams is shown in Figure 1.

Gamma Knife treatment planning

**Figure 2:**
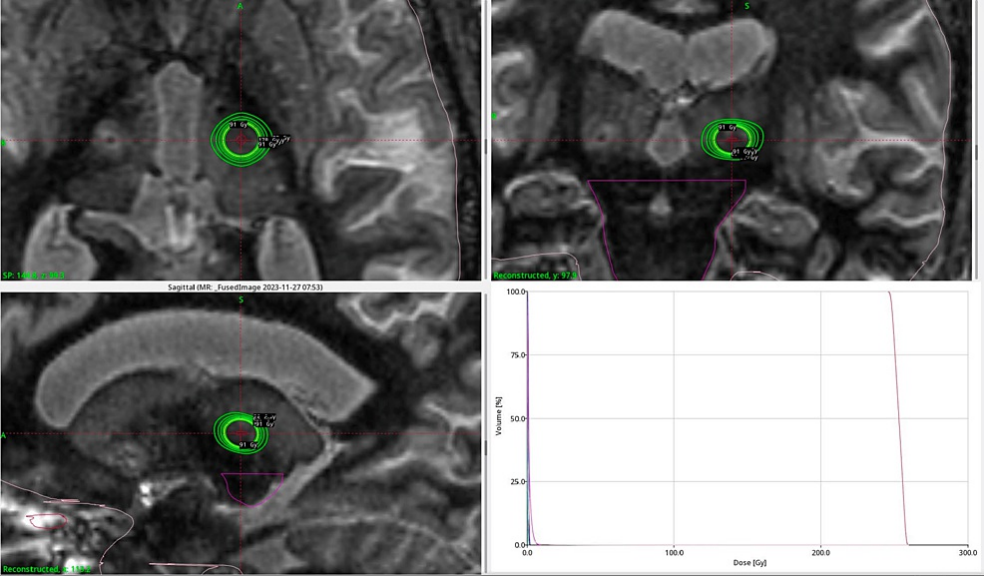
Gamma Knife (GK) treatment plan for essential tremor utilizing a 4 mm collimator.

An unblocked 4 mm isocenter was centered on the VIM target using Leksell GammaPlan v11.3.1 and the Gamma Knife Icon, an SRS device that employs 192 intersecting Co-60 beams, whose average photon energy is 1.25 MeV. The TMR10 dose calculation algorithm was used. The Gamma Knife Icon is dosimetrically identical to its predecessor, the Gamma Knife Perfexion, and its successor, the Esprit. The resulting treatment plan shown in Figure 2 delivered 91 Gy to the 70% isodose line.

Gyroscopic radiosurgery treatment planning

A gyroscopic radiosurgery treatment plan was generated for the ZAP-X® radiosurgical robot. Of note, this device gyroscopically rotates a LINAC around a target isocenter, cross-firing its 3MV beam with a solid angle dispersion of more than 2.0π [13].

**Figure 3:**
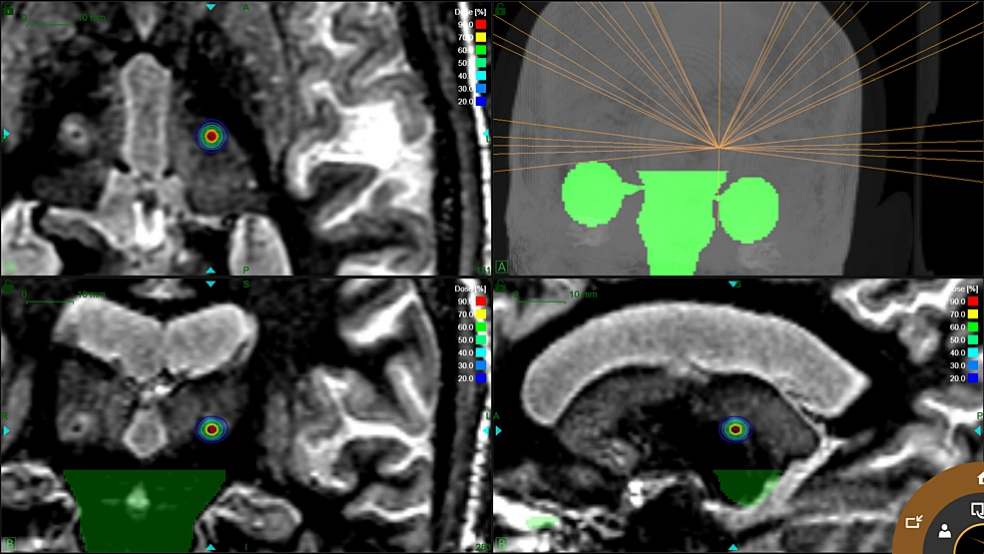
Gyroscopic radiosurgery (GRS) treatment plan for essential tremor using a 3 mm collimator.

Once again, as described above in “Target selection”, the patient images (axial CT and MRI), in which VIM had been delineated were used for treatment planning delivering in a single isocenter 91 Gy to the 70% isodose line (Dmax = 130 Gy). Two separate plans were generated with 3 mm and 4 mm collimators. Both plans are shown in Figure 3. Although fully tested and validated, it should be noted that the 3 mm collimator is not yet commercially available (Sabe, Alders, Klassen et al. submitted for publication).

Dosimetry

Having shown treatment plans above (Figures 1-3), here we summarize the key dosimetric parameters for the different radiosurgery platforms. The volume receiving 5 Gy (V5Gy), 10 Gy (V10Gy), and 12 Gy (V12Gy ) is reported as well as the Gradient Index (GI) [14] (V35%/V70%), as well as V70%, and V35%, or those volumes receiving prescription dose and half of prescription dose. These parameters which are summarized in Table 1 allow us to make uniform comparisons across the three platforms.

**Table 1:** Summary of Dosimetric Parameters GK: Gamma Knife, RRS: robotic radiosurgery, GRS: gyroscopic radiosurgery

Parameters	GK (4mm-1.25MeV)	RRS (5mm-6MV)	GRS (4mm-3MV)	GRS (3mm-3MV)
Beams	192	153	36	36
V_5Gy_ (cm^3^)	4.66	9.43	6.30	2.29
V_10Gy _(cm^3^)	1.54	3.06	1.25	0.59
V_12Gy_ (cm^3^)	1.16	2.28	0.904	0.44
V_35%_ (mm^3^)	172	480	143	47.7
V_70%_ (mm^3^)	50	88.9	33	11.3
Gradient Index (V_35%_/V_70%_)	3.44	5.40	4.33	4.22

The discreet peaks of the physical energy at 1.17MeV and 1.33MeV of the GK system are considered approximately equivalent to the 3MV accelerating potential of the GRS, originating from a photon Bremsstrahlung spectrum with similar effective energy. The mean energy of the ZAP-X photon beam was determined to be 1.15MeV.

## Discussion

In this study we report the theoretical technical aspects of treating ET across three different radiosurgical platforms. Of these three, the GK is the established historical “gold standard” for treating ET with several studies reporting its safety and efficacy [8-10]. Notably, RRS, and the newest SRS technology, GRS, are both deemed safe and effective for the treatment of brain tumors and have the necessary regulatory clearance to perform thalamic lesioning [15,16]. The current analysis focused on the specific dosimetric properties that are deemed most appropriate for “lesioning” a small target like the VIM nucleus of the thalamus, i.e. V5Gy, V10Gy, V12Gy, Gradient Index, V70% and V35%.

When lesioning functional brain targets with SRS, arguably the single most important determinant of plan quality is collimator size. In this study, collimators ranged from 5 mm for the RRS device, to 4 mm for the GK, with GRS capable of using both 3 and 4 mm collimators. Collimator diameter heavily (but not exclusively) influences the amount of normal brain external to the target that is irradiated. This effect is best appreciated by comparing overall irradiated brain volumes at all dose levels (12Gy, 10Gy, and 5Gy), with, for example, the V12Gy of the 5 mm collimator RRS plan being more than five times larger than that of the GRS system using a 3 mm collimator. Besides collimator diameter, these volumes are also determined by beam energy, collimator leakage, and the effect of SAD on beam penumbra. Given its known association with symptomatic radionecrosis in SRS-treated AVM and brain metastases, V12Gy is of special relevance. It is a radiosurgical axiom that the probability of radiation-induced side effects invariably increases with the volume of irradiated brain. Meanwhile, the V5Gy of the RRS is largest while that of the GRS is smallest. This is expected given that GRS has the lowest beam energy, the largest collimator shielding, and the smallest field size.

The GI, a metric to quantitatively describe the peripheral dose fall-off, was evaluated in our study. The results show that the GK has the lowest GI followed by GRS and then RRS. However this should be interpreted with caution since the actual target volume being lesioned (VIM) is so small, questioning the clinical relevance here of GI.

The V35% (volume receiving 50% of prescription dose) was also determined for all three platforms. Notably this value for the GRS 3 mm collimator is nearly three times lower than that for GK and almost an order of magnitude lower than RRS. This again is related to the small size of the 3 mm collimator soon to be available with the GRS system. The V70% was also determined for the three platforms and shows a similar profile to the V35% with the GRS plan having the smallest value. This again is attributed to utilization of the 3 mm collimator.

Although generally safe, there are risks to treating ET with SRS, with some patients demonstrating ‘hyperesponder’ behaviour. The exact cause of such a response is not known, but likely stems from extraneous radiation landing outside the target region (VIM), thereby affecting the adjacent thalamus [17]. Injury to the nearby internal capsule is another such well reported complication, seemingly also made more likely by “spillage” of radiation outside the VIM nucleus [17]; approximately 4-8% of patients will experience contralateral motor symptoms ranging from numbness to tingling to frank weakness and/or permanent disability [8]. These very real risks from what appears to be a safe non-invasive operation underline the importance of the present study. When lesioning functional brain targets with SRS, the more concentrated the radiation, i.e. the sharper the knife, theoretically the safer the procedure.

Inducing a clinical effect by irradiating a small volume of brain tissue requires a remarkably high dose. Treatment for obsessive-compulsive disorder (OCD) using bilateral, single, 4 mm collimators at a dose of 180Gy was often ineffective and paired isocenters were required to achieve adequate response rates [18]. This effect is mirrored in animal studies where an increase in the length of rat spinal cord from 2 mm to 4 mm to 2 mm decreased the ED50 for myelopathy from 87.8Gy to 53.7Gy [19]. While the dose for ET has been empirically titrated using 4 mm collimator-based treatments to around 120-130Gy, it would be expected that a higher dose would be required if a significantly smaller volume was irradiated. As a consequence, determining the optimal dose and volume remains a work in progress. Nevertheless, the ability to be able to focus radiation within a smaller volume of tissue may yield advantages for future functional treatments.

Finally, it is worth emphasizing that the present-day biological basis for understanding radiosurgical VIM thalamotomy may be seriously flawed. There is growing recognition of the phenomenon of radiomodulation, whereby small-volume radiation can change brain function without lesioning. Both upregulation and downregulation of local brain circuits have been demonstrated in animal models with doses as low as 10Gy [20]. In light of these new findings, it is worth being mindful of the potential effects of lower radiation doses to highly eloquent thalamic structures which to date have remained totally unaccounted for in clinical studies. Clearly more research is warranted.

## Conclusions

In this technical note we have compared the dosimetric profile of three major stereotactic radiosurgery platforms (GK, RRS, and GRS) for the treatment of ET. In terms of pure dosimetric profiles, GRS has the smallest focus, which may be advantageous for treating essential tremor. This is largely due to the small collimator sizes and low beam energy. It should be emphasized that this is purely a technical study and that future work should test the clinical efficacy of each platform for the treatment of ET. It could be postulated that since GRS utilizes a smaller collimator, its clinical efficacy with lower side effect profile may be superior to the other platforms. Clinical studies are very much needed to corroborate such a hypothesis.
